# Income and education affect prognosis and treatment in symptomatic myeloma: A population-based study on 8672 multiple myeloma patients diagnosed 2008–2021 from the Swedish myeloma registry

**DOI:** 10.1007/s00277-025-06214-3

**Published:** 2025-01-24

**Authors:** Gunnar Larfors, Kristina Carlson, Christopher Day, Sigrun Einarsdottir, Gunnar Juliusson, Moshtaak Karma, Dorota Knut-Bojanowska, Ingigerður Sólveig Sverrisdóttir, Ingemar Turesson, Mariana Villegas-Scivetti, Cecilie Hveding Blimark

**Affiliations:** 1 Unit of Haematology, Department of Medical Sciences, Uppsala University, Uppsala, Sweden; 2 Department of Medicine and Clinical Nutrition, Institute of Medicine, Sahlgrenska Academy, University of Gothenburg, Gothenburg, Sweden; 3 Department of Haematology and Coagulation, Sahlgrenska University Hospital, Gothenburg, Sweden; 4 Department of Laboratory Medicine, Stem Cell Center, Lund University and Department of Haematology, Skåne University Hospital, Lund, Sweden; 5 Department of Haematology, Södra Älvsborg Hospital, Borås, Sweden; 6 Department of Haematology, Uddevalla Hospital, Uddevalla, Sweden; 7 Department of Medicine, University of Iceland, Reykjavik, Iceland; 8 Department of Haematology, Skåne University Hospital Malmö, Lund, Sweden

**Keywords:** Multiple myeloma, Survival, Socioeconomic factors, Epidemiology, Cohort studies

## Abstract

Despite advancements in multiple myeloma treatment, prognostic variability persists. We investigated the impact of income and education on treatment and survival in a country with publicly funded healthcare. We analysed data from the Swedish Myeloma Registry (2008–2021) linked to national registers. Cox models assessed survival, adjusting for demographics and comorbidities. Treatment patterns were compared using cumulative incidence functions. Among 8,672 patients, higher education and income correlated with prolonged survival. Adjusted hazard ratios (HRs) for low income were 1.4 (95% CI 1.3–1.5) and for low education were 1.3 (95% CI 1.2–1.4). Higher income patients were more likely to receive lenalidomide (HR 1.5, 95% CI 1.3–1.6) and pomalidomide (HR 1.7, 95% CI 1.4-2.0), and less likely to receive melphalan tablets (HR 0.8, 95% CI 0.7–0.9). Low-income patients were less likely to undergo stem cell transplant (HR 0.8, 95% CI 0.7–0.9). Immigrant status or biological sex did not influence outcomes. Even in a tax-funded system, socioeconomic disparities impact myeloma survival and treatment. Lower socioeconomic status correlates with inferior outcome and more conservative treatment. Attitudinal biases may contribute to these disparities. Better treatment for the less privileged patients could significantly improve myeloma survival, advocating for efforts to overcome the influence of socioeconomic status.

## Introduction

Myeloma prognosis has seen a long awaited improvement in the last two decades, following the introduction of new treatment modalities [1]. Although a majority of patients can now live more than five years, the prospects of longevity are highly variable between newly diagnosed patients. This variation cannot be explained by differences in disease characteristics alone. Bone marrow involvement, end organ damage and genetic aberrations all influence prognosis, but patient characteristics, such as age, comorbidities and frailty are equally important [2, 3]. A link between socioeconomic factors and prognosis has gained attention during recent years [4–6], but most studies derive from populations with unequal access to expensive treatments. We investigated whether income and education influence modern myeloma treatment choice and survival also in a country where nearly all health care is publicly funded and accessible for all.

## Materials and methods

### Study population

The study base included all Swedish residents who were newly diagnosed with symptomatic myeloma and registered in the Swedish Myeloma Registry. The cohort included all patients diagnosed between 2008, when the register was initiated, and 2021. In all, the cohort included 8,672 patients. For comparison, four controls to each case were randomly selected from the general population. Controls were matched on age, sex and region of residence and had to be alive at the time of diagnosis of the case.

### Data sources

All Swedish residents are assigned an individual number at birth or immigration, which is then used for all registered contacts with authorities. Consequently, most Swedish registries can be combined. For this study, the Swedish Myeloma Registry was linked to The Prescribed Drug Register, The Patient Register and the Cancer Register (all of which are held by the National Board of Health and Welfare), and further to the Population Register and The Longitudinal Integrated Database for Labour Market Research (LISA) from Statistics Sweden.

LISA is a yearly compilation of data from different sources on educational background, income, sick leave, household size, and several other socioeconomic indicators for all Swedish residents 16 years of age and older from 1990 onwards [7]. Data on disposable income include not only taxable income (such as salary, dividends, capital gain, interests, pensions), but also all types of governmental benefits (health insurance, child support, study allowances and similar). Since tax-deductible losses in the capital market, from property sales, or from interests are also included, income can in extreme cases be negative. To avoid undue effect of outliers, personal and family income were grouped into highest quartile, lowest quartile and those in-between. Education level was divided into three categories; primary school only (≤ 9 years), secondary education (10–12 years), or academic education (≥ 13 years).

Data on stem cell transplants (HSCT) were retrieved from the 1-year follow-up form in the myeloma register. Data on lenalidomide, pomalidomide, and melphalan treatment were retrieved from the Swedish Prescribed Drug Register. During the years studied, lenalidomide and pomalidomide were covered by patents and represented new and expensive treatment, whereas melphalan tablets served as an example of an older, low-cost treatment. Data on comorbidities were retrieved from the Swedish Patient Register and the Swedish Cancer Register, and the Charlson comorbidity index was constructed based on diagnoses registered within five years preceding myeloma diagnosis. To construct the Charlson comorbidity index from registry data, the Royal College of Surgeons’ method was used [8]. Patients were followed in the Swedish Population Register until death, permanent emigration or 31st December 2022.

To determine whether myeloma patients with lower socioeconomic status had higher mortality due to other causes than myeloma, causes of death were collected from the Swedish Cause of Death Register, which includes data from death certificates from 1947 onwards for all residents in Sweden [9].

### Statistical analysis

Analyses on overall survival were performed with Cox proportional hazards models, yielding hazard ratios (HR) with 95% confidence intervals (95% CI) as measures of relative risk of death [10]. Differences in prescriptions were analysed with cumulative incidence functions with death as a competing event, using Gray’s test to assess equivalence between groups [11]. To address confounding, the same differences were also addressed by adjusted cause-specific hazard regression. For the analysis of predictors for HSCT treatment multi-variable Poisson regression was used [12]. All p-values were two-sided and a value below 0.05 was considered statistically significant. All analyses were performed with SAS 9.4 statistical software (SAS Inc., Cary, NC).

## Results

The baseline characteristics of the cohort are outlined in Table 1.

**Table 1:** Baseline characteristics at symptomatic myeloma diagnosis and their impact on overall mortality among 8672 patients. (CI = confidence interval, ISS = International Staging System)

		*n*	%	Hazard ratio of death with 95% CI	*P*
Age	< 65	2273	26	Ref	-
	65–80	4590	53	2.3 (2.1–2.5)	< 0.0001
	> 80	1809	21	6.1 (5.6–6.6)	< 0.0001
Sex	Female	3690	43	Ref	-
	Male	4982	57	1.0 (1.0-1.1)	0.12
Year	2008–2012	2833	33	Ref	-
	2013–2017	3198	37	0.8 (0.8–0.9)	< 0.0001
	2018–2021	2641	30	0.7 (0.7–0.8)	< 0.0001
Charlson comorbidity index	0	5380	62	Ref	-
	1	2029	23	1.5 (1.4–1.6)	< 0.0001
	≥ 2	1263	15	2.4 (2.2–2.5)	< 0.0001
ISS risk class	Low	1241	20	Ref	-
	Intermediate	2716	44	1.7 (1.5–1.8)	< 0.0001
	High	2226	36	2.7 (2.5-3.0)	< 0.0001
	*Missing*	*2489*			
Haemoglobin level at diagnosis (g/dl)	> 10.0	5727	66	Ref	-
	< 10.0	2913	34	1.5 (1.4–1.6)	< 0.0001
	*Missing*	*32*			
Creatinine level at diagnosis (µmol/l)	< 177	7205	83	Ref	-
	> 177	1430	17	1.5 (1.4–1.6)	< 0.0001
	*Missing*	*37*			

As expected, the majority of patients were male, and approximately three out of four were above age 65 at diagnosis. Median and mean follow-up was 3.1 years and 3.9 years, respectively. Median survival was estimated to 4.2 years; 8.8 years among patients below 65 years of age at diagnosis, compared to 3.3 years among older patients. In 8.7% of patients, the diagnosis of symptomatic myeloma had been preceded by a known smouldering myeloma or solitary plasmocytoma, this proportion did not vary across socioeconomic groups.

Socioeconomic characteristics were similar in the myeloma cohort compared to controls, matched on age, sex, and region of residence (Table 2).

**Table 2:** Indicators of socioeconomic status among myeloma patients and comparators and their relationship to overall mortality in myeloma patients. Hazard ratios of death are adjusted for age, sex, year of diagnosis and comorbidity index. (EU = European Union, CI = confidence interval)

		Patients (%)	Comparators (%)	Adjusted hazard ratio of death with 95% CI	*P*
Personal income	Top quartile	2250 (26)	8611 (25)	Ref	-
	Intermediate	4225 (49)	17,116 (50)	1.2 (1.1–1.3)	< 0.0001
	Lowest quartile	2136 (25)	8771 (25)	1.3 (1.2–1.4)	< 0.0001
	*Missing*	*61*	*63*		
Household income	Top quartile	2321 (27)	8632 (25)	Ref	-
	Intermediate	4286 (50)	17,222 (50)	1.2 (1.1–1.3)	< 0.0001
	Lowest quartile	2004 (23)	8644 (25)	1.4 (1.3–1.5)	< 0.0001
	*Missing*	*61*	*63*		
Education (years)	≥ 13	2245 (26)	8570 (25)	Ref	-
	10–12	3551 (42)	14,045 (41)	1.2 (1.1–1.3)	< 0.0001
	≤ 9	2730 (32)	11,490 (34)	1.3 (1.2–1.4)	< 0.0001
	*Missing*	*146*	*456*		
Country of birth	Sweden	7651 (88)	30,125 (87)	Ref	-
	Nordic country except Sweden	370 (4)	1495 (4)	1.1 (1.0-1.2)	0.2
	EU except Nordic countries	221 (3)	1107 (3)	1.2 (1.0-1.4)	0.06
	Rest of the world	429 (5)	1832 (5)	1.0 (0.9–1.2)	0.6
	*Missing*	*1*	*2*		
Living alone	No	5285 (61)	20,222 (59)	Ref	-
	Yes	3326 (39)	14,276 (41)	1.2 (1.1–1.3)	< 0.0001
	*Missing*	*61*	*63*		
University hospital region	No	3241 (37)	12,931 (37)	Ref	-
	Yes	5431 (63)	21,630 (63)	1.0 (0.9-1.0)	0.6

Among patients, median survival was doubled in those with higher education, compared with patients with primary school only (6.0 years vs. 3.0 years). Similarly, patients in the highest family income quartile had a median survival of 7.5 years, compared to 2.8 years among patients from the lowest quartile. The survival differences were to some degree explained by differences at baseline, in particular age at diagnosis. Patients with shorter education or from low income households were on average older; in the highest income group mean age at diagnosis was 65 years, compared to 72 years in the middle group and 75 years in the lowest income group. The less affluent patients also had more comorbidities, and did more often have high ISS risk class, anaemia and renal impairment at diagnosis. In a multivariable proportional hazards regression, with adjustments for age, sex, year of diagnosis and comorbidity index, patients from low income households had 40% higher risk of death than those from high income households, and patients with primary school only had 30% higher risk of death compared to patients with higher education, *p* < 0.0001 for both analyses (Table 2). Patients living alone were also at higher risk of death during follow-up. Further adjustment for ISS risk class, anaemia or renal impairment at diagnosis did not alter these results. Patient sex, country of birth, or whether the patient lived in one of the university hospital regions did not change prognosis.

To determine potential effect modification by sex, the same analyses were performed separately for male and female patients. No significant effect modification could be detected, although men from the lowest family income quarter had 52% higher risk of death than men from the highest family income quarter, whereas women from the lowest income quarter only had 27% increased risk of death compared to women from the highest income quarter. This interaction almost reached statistical significance (*p* = 0.08). Regarding personal income and education, the mortality risk increases were similar in men and women.

Among matched comparators from the general population, socioeconomic indices were also associated with survival. Following adjustments for age, sex and comorbidities, the hazard ratio of death was 80% higher in the lowest household income group compared to the highest (HR = 1.8, 95% CI 1.7-2.0), and 50% higher in comparators with primary school only compared to those with higher education (HR = 1.5, 95% CI 1.4–1.6). Consequently, the relative difference in survival between patients and comparators was lowest among the least affluent, where the comparators had the highest background mortality. On the other hand, the higher mortality in myeloma patients with low socioeconomic status did not seem to stem from other causes than myeloma. Comparing causes of death, 83% of the myeloma patients from the lowest household income group and 81% of patients from the highest household income group had myeloma listed as cause of death (*p* = 0.5).

**Fig. 1:**
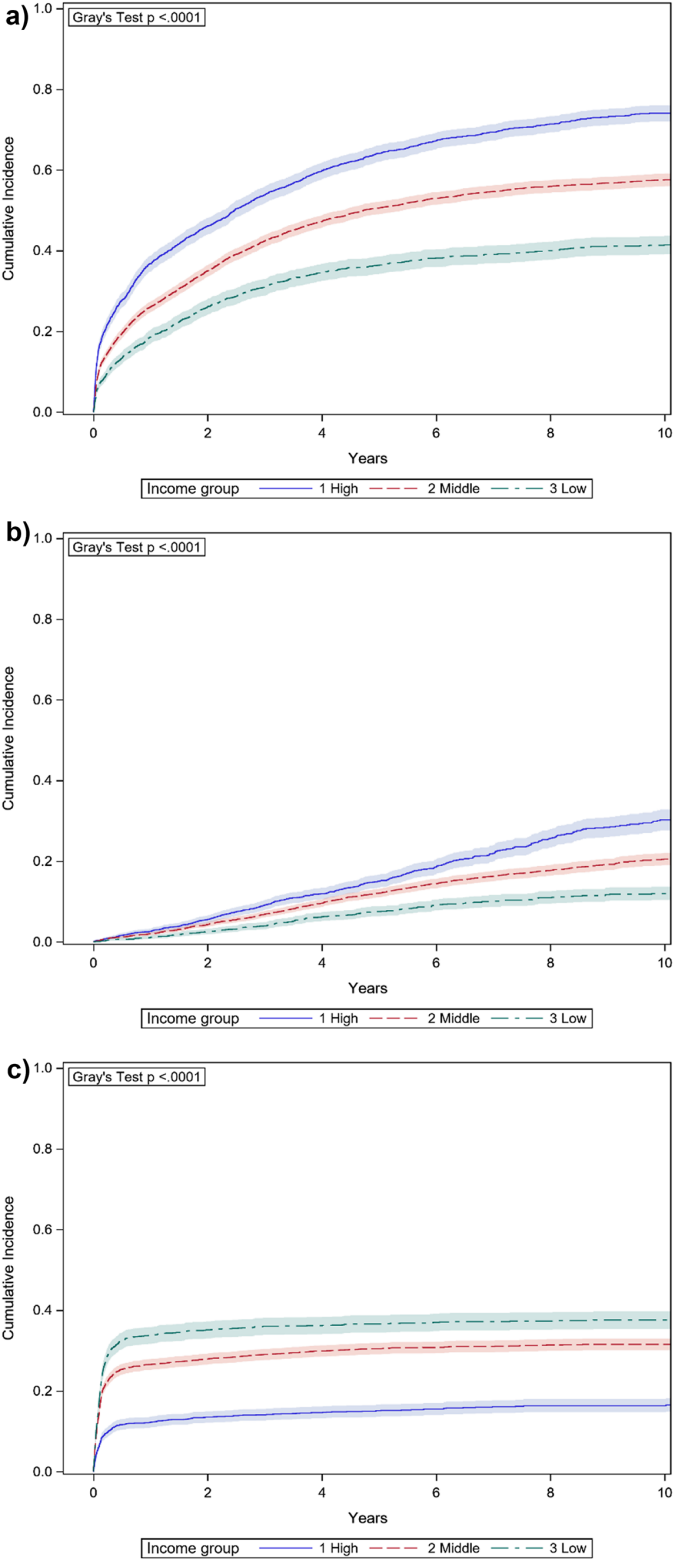
a-c. Cumulative incidence function plots of prescribed lenalidomide (**a**), pomalidomide (**b**), and oral melphalan (**c**), divided on household income level (with 95% confidence intervals)

Considering prescription patterns, patients with the highest family incomes were also more likely to have been treated with a lenalidomide containing regimen, or a pomalidomide containing regimen, while patients with the lowest family incomes were more often treated with melphalan-prednisone based regimens (Fig. 1).

Following adjustment for age and year of diagnosis, patients in the highest income group were 50% more likely to be treated with lenalidomide, 70% more likely to be treated with pomalidomide and 20% less likely to be treated with melphalan tablets compared to patients from the lowest income group (lenalidomide HR 1.5, 95% CI 1.3–1.6; pomalidomide HR 1.7, 95% CI 1.4-2.0; melphalan HR 0.8, 95% CI 0.7–0.9).

Among myeloma patients below 65 years of age at diagnosis, patients with low income or with less education were also less likely to be treated with a stem cell transplant (Table 3).

**Table 3:** Probability of receiving a stem cell transplant in the first year after diagnosis among myeloma patients < 65 years, divided on socioeconomic indicators. Risk ratios estimate the relative probability of a stem cell transplant after adjustment for age, sex, year of diagnosis and comorbidities. (HSCT = hematopoietic stem cell transplant, CI = confidence interval)

		HSCT performed yes/no	%	Adjusted risk ratio (95% CI)	*P*
Personal income	Top quartile	814/140	85	Ref	-
	Intermediate	610/171	78	0.9 (0.9-1.0)	0.005
	Lowest quartile	220/115	66	0.8 (0.8–0.9)	< 0.0001
	*Missing*	5/5			
Household income	Top quartile	854/158	84	Ref	-
	Intermediate	632/165	79	1.0 (0.9-1.0)	0.1
	Lowest quartile	158/103	61	0.8 (0.7–0.9)	< 0.0001
	*Missing*	5/5			
Education (years)	≥ 13	613/97	86	Ref	-
	10–12	759/205	79	0.9 (0.9-1.0)	0.001
	≤ 9	261/119	69	0.9 (0.8–0.9)	< 0.0001
	*Missing*	16/10			
Country of birth	Sweden	1402/321	81	Ref	-
	Nordic country except Sweden	60/16	79	1.0 (0.8–1.1)	0.4
	EU except Nordic countries	26/10	72	0.9 (0.8–1.1)	0.4
	Rest of the world	157/37	81	1.0 (0.9–1.1)	0.7
Living alone	No	1204/235	84	Ref	-
	Yes	436/145	75	0.9 (0.9-1.0)	0.0003
	*Missing*	5/4			

Even among patients less than 65 years of age at diagnosis with no recorded comorbidities, 29% in the low income group had no recorded stem cell transplant, compared to 12% in the high income group.

While both prognosis and treatment were altered in patients with less education, from low income households or living alone, being born outside of Sweden did not seem to impact neither treatment options nor outcome (Tables 2 and 3).

## Discussion

From our population-based data, we can demonstrate that education and private economy are correlated with survival in multiple myeloma, even in a country where the cost of health care is covered almost completely by taxes. Although patients with lower socioeconomic status were generally older, had more comorbidities, and had more often anaemia or renal impairment at diagnosis, survival differences remained after adjustment for these potential confounders.

Furthermore, in spite of their higher risk of death, and in spite of worse prognostic factors at diagnosis, our data suggest that patients from lower socioeconomic conditions are treated more conservatively than patients from more affluent homes. We did not have complete data on all treatments the patients had received, but judging from prescription patterns, patients from low income homes seem to be treated more often with older, less effective drugs. Patients with lower income were also to a lesser degree treated with a stem cell transplant, perhaps the most obvious sign of intensive myeloma first-line treatment. It should be noted that this analysis only included patients under 65 years of age at diagnosis, ages where few Swedes are retired and income usually reflects employment conditions.

The last years has seen a number of studies demonstrating better survival among myeloma patients from more affluent homes or neighbourhoods. Most reports come from the US and are based on large-scale data from the National Cancer Database or SEER (Statistics, Epidemiology and End Results) [6, 13–18]. As opposed to most European countries, these results may reflect differences in insurance policies [19]. A study on SEER data from 2018 described differences both in treatment and outcome based on which Medicare plan the patients were covered by [20]. For example, prescription drugs like lenalidomide and pomalidomide were not covered for a significant part of the population, resulting in more frequent use of traditional chemotherapy. The study also showed that health insurance coverage correlated to socioeconomic conditions.

Socioeconomic conditions among myeloma patients have also been investigated in a number of smaller studies from other parts of the world. Studies from Australia [21] and New Zealand [22] have demonstrated lower survival in deprived areas. A Greek single-centre cohort of 223 patients demonstrated better survival in patients with higher socio-economic status [23]. In a Chinese cohort of only 85 patients, a significant difference in median survival could be demonstrated between patients from low and high socioeconomic status [24]. There is also a previous Swedish study demonstrating survival differences in myeloma patients based on occupational status, but this study was based on patients diagnosed before the introduction of modern myeloma therapy [25].

Interestingly, low education has been linked to worse prognosis in a Chinese study [26]. Meanwhile, a recently published Danish study did not observe a prognostic impact of completion of tertiary level education in myeloma patients [27].

Unlike previously reported results [6, 15, 28, 29], residents in the seven regions with academic medical centres did not do better than patients from the more rural fourteen regions in Sweden. Our study was, however, not specifically intended at this research question. We did not study at which specific hospital the individual patients received their treatment, and did not analyse differences in treatment patterns between hospitals.

A hypothetical explanation to socioeconomic differences in Swedish myeloma care would be that immigrants from poorer countries generally have lower socioeconomic status and might have language and cultural barriers hampering their access to care. However, judging from our data, immigrant status does not explain the differences. Patients born outside the European Union had the same prognosis and were equally often treated with stem cell transplants as were Swedish born patients. Neither the outcome nor treatment pattern seemed to be influenced by patients’ sex.

Instead, the observed differences might be driven by the attitudes towards treatment that influence both patients and doctors. In an earlier study on Swedish chronic myeloid leukaemia patients, no differences in survival and treatment were demonstrated, other than what could be explained by baseline health status [30]. In comparison, chronic myeloid leukaemia is today a disease with a fairly uniform treatment where the vast majority is treated with first-line tyrosine kinase inhibitors. Myeloma, on the other hand, is still a highly fatal disease where almost all patients are faced with repeated choices of treatment modalities. This decision-making process provides personalised care, but may also be vulnerable to biased opinions based on patient background.

Our study provides the first large-scale population-based myeloma cohort with individual level data on income and education, and set in a country with uniform health care funding. With more than 8,000 patients, it is powered to rule out chance as explanation to the results, and disparities in therapy could not be attributed to differences in insurance policies. However, the study has obvious limitations. We had incomplete data on given therapies, especially in later lines. Hence, the true variation in therapy across socioeconomic groups may be larger or smaller than what we could deduct from prescription patterns and records of stem cell transplants. Further, given the historical nature of data, modern risk prognostication with data on chromosomal abnormalities was only available for a minority of patients. Nevertheless, we have no reason to believe that chromosomal abnormalities would depend on socioeconomic status.

In conclusion, our results indicate that both treatment and survival in myeloma depend on income and education. Myeloma survival could be increased with more than a year if we achieved the same results for all patients as we do with the ones in the wealthiest and most educated quartile.

## Data Availability

Due to legislative restrictions, data from Swedish national patient databases are not shared openly. The data can be made available upon reasonable request to the corresponding author.
